# Tissue-resident immunity in the lung: a first-line defense at the environmental interface

**DOI:** 10.1007/s00281-022-00964-2

**Published:** 2022-10-28

**Authors:** Dimitra E. Zazara, Ioannis Belios, Jöran Lücke, Tao Zhang, Anastasios D. Giannou

**Affiliations:** 1 Division for Experimental Feto-Maternal Medicine, Department of Obstetrics and Fetal Medicine, University Medical Center of Hamburg-Eppendorf (UKE), Martinistr. 52, 20246 Hamburg, Germany; 2 University Children’s Hospital, UKE, Hamburg, Germany; 3 Department of General, Visceral and Thoracic Surgery, UKE, Hamburg, Germany; 4 Section of Molecular Immunology and Gastroenterology, I. Department of Medicine, UKE, Hamburg, Germany

**Keywords:** Tissue-resident immunity, Lung, Infections, Asthma, Cancer, Metastasis

## Abstract

The lung is a vital organ that incessantly faces external environmental challenges. Its homeostasis and unimpeded vital function are ensured by the respiratory epithelium working hand in hand with an intricate fine-tuned tissue-resident immune cell network. Lung tissue-resident immune cells span across the innate and adaptive immunity and protect from infectious agents but can also prove to be pathogenic if dysregulated. Here, we review the innate and adaptive immune cell subtypes comprising lung-resident immunity and discuss their ontogeny and role in distinct respiratory diseases. An improved understanding of the role of lung-resident immunity and how its function is dysregulated under pathological conditions can shed light on the pathogenesis of respiratory diseases.

## Introduction

Located at the environmental interface, the lung constantly encounters external insults threatening host homeostasis. The multifaceted protective function of the lung relies on a delicate balance among repelling invading pathogens while in parallel tolerating harmless particulate matter and sustaining its vital function. A pivotal safeguard of this balance is the local lung tissue microenvironment consisting of the respiratory epithelium and a sophisticated network of non-circulating lung-resident immune cells [1].

In the past decade, several studies shed light on tissue-resident immunity and went beyond the “strict limits” between innate and adaptive immunity. It is now well known that, apart from circulating immune cells, which are activated upon antigen encounter by the first line of defense and then migrate to the site of inflammation, there are also innate and adaptive immune cells with protective properties, which reside in the tissue, respond fast, and effectively cope with every invading pathogen [2]. Tissue-resident cells extend across adaptive and innate immunity and, as integral part of an immune sensing network, provide first-line tissue-specific immune protection. Despite its protective function, mounting evidence also highlights the role of derailed tissue-resident immunity in disease pathogenesis. Although the role of the adaptive tissue-resident immunity in sustaining respiratory health has been significantly appraised [3, 4], innate and innate-like immune cell subtypes are also important in this context [5]. In this review, we focus on the main innate, innate-like and adaptive lung-resident immune subsets and discuss their ontogeny and role in respiratory health and disease.

## Tissue-resident innate immune cells in pulmonary homeostasis

### Macrophages, key orchestrators of respiratory immunity

**Fig. 1:**
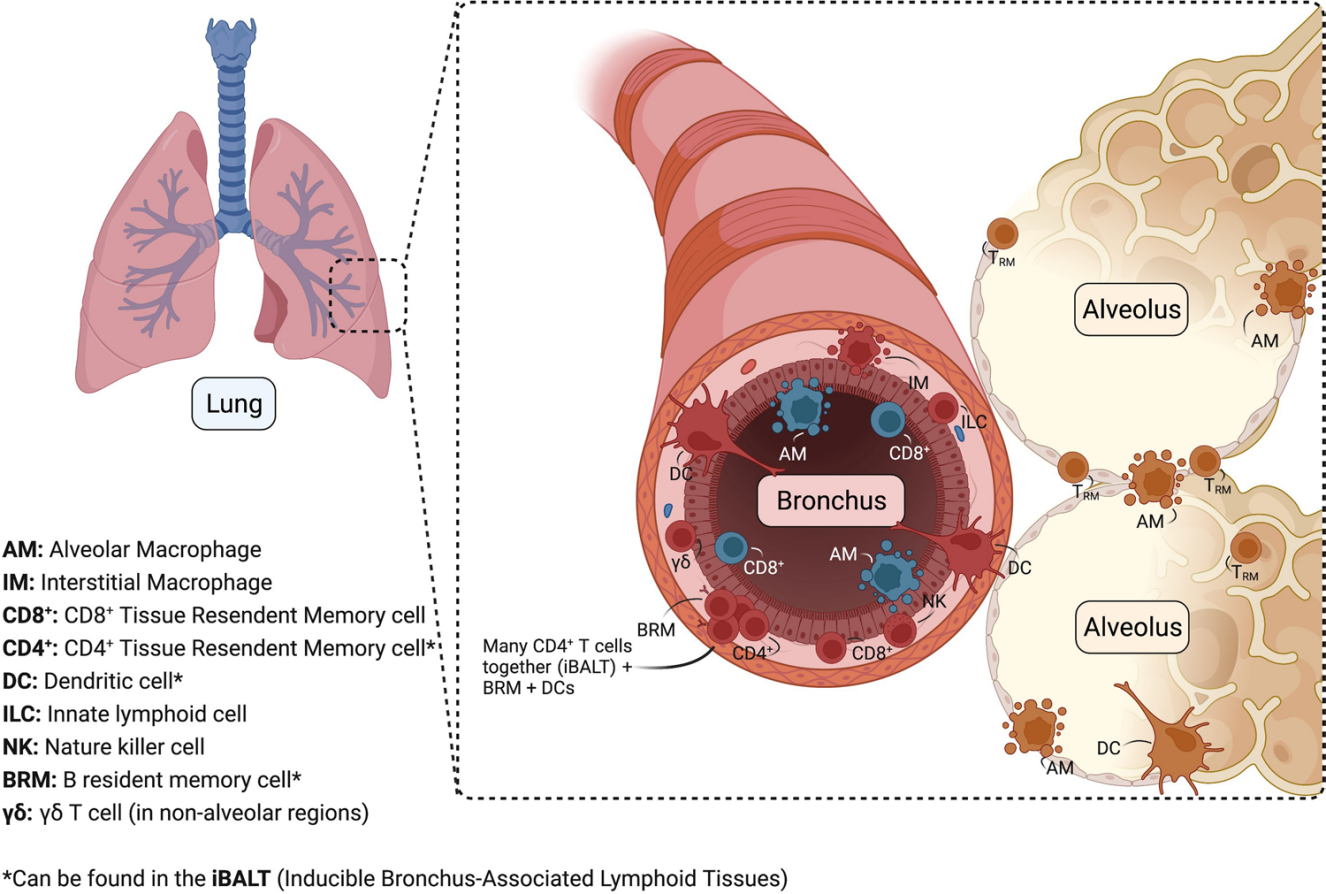
Location of immune tissue-resident cells in the lungs (alveoli and bronchus). Created with BioRender.com

Lung-resident macrophages comprise the alveolar (AM) and interstitial (IM) macrophages [5–7]. AM are the majority of lung-resident macrophages and are located in the alveolar space thereby constantly facing external stimuli, while IM are fewer and can be found within the lung parenchyma [7] (Fig. 1). AM interact closely with the respiratory epithelium and remove invading pathogens and other particles [5] through phagocytosis.

**Fig. 2:**
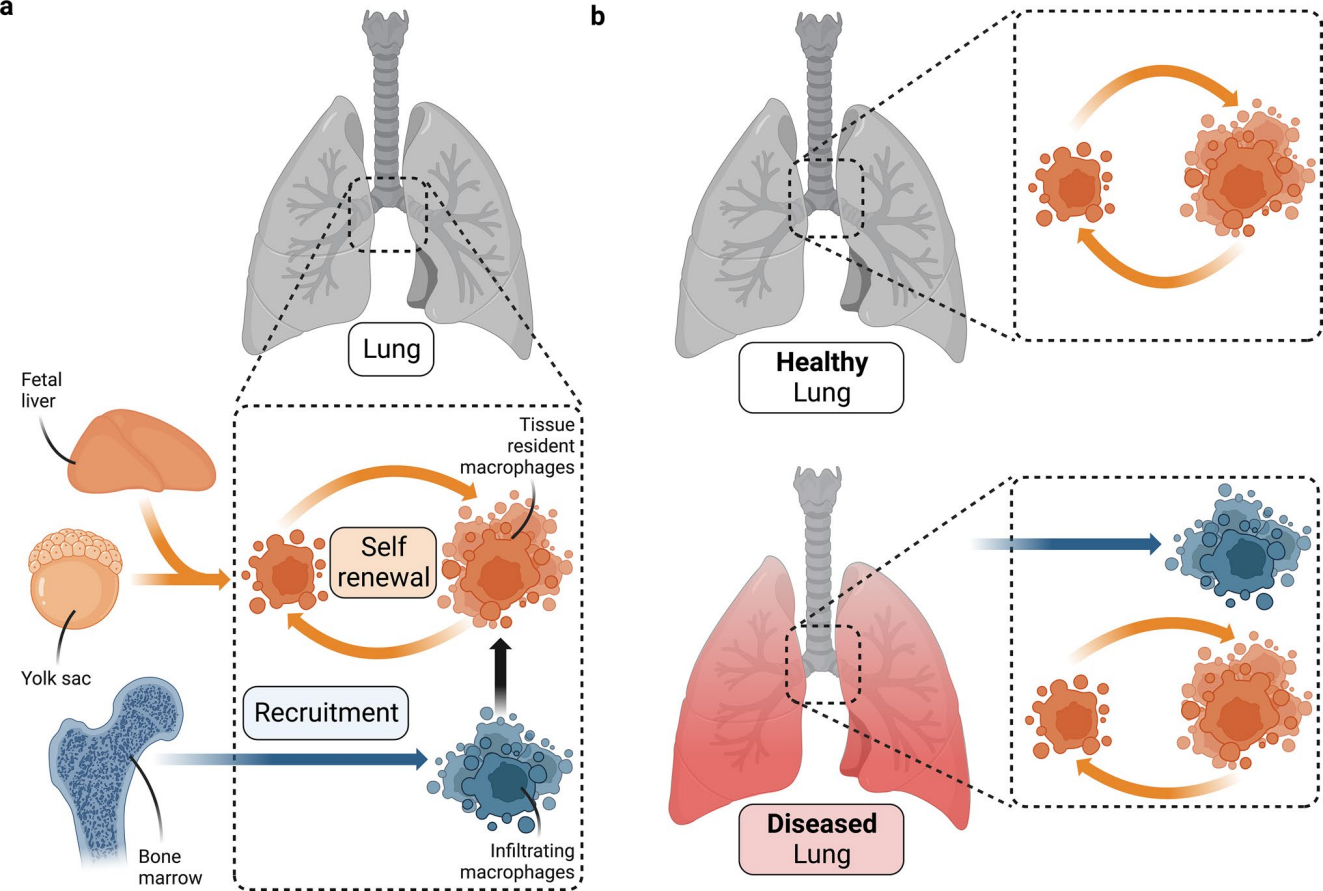
Fetal emergence and life-long maintenance of alveolar macrophages. Origin and mechanisms for **a** lungresident macrophage replenishment **b** in a healthy or diseased lung

Lung-resident macrophages arise independently either from embryonic progenitor cells or circulating monocytes (Fig. 2) [6, 8, 9]. Studies in mice highlight a fetal origin of AM in steady state, which emerge as the end-products of the postnatal maturation of fetal liver- and yolk sac-derived monocytes residing in the lung, in response to transforming growth factor β (TGF‐β), cytokine granulocyte–macrophage colony‐stimulating factor (GM‐CSF) and peroxisome proliferator‐activated receptor γ (PPARγ) [9–12]. The life-long maintenance of AM relies on their local self-expansion and is largely independent of circulating monocytes in steady state (Fig. 2a, b) [10, 11]. However, augmented contribution of circulating monocytes to AM replenishment is observed with aging and upon respiratory infections, lung fibrosis, or regeneration (Fig. 2b) [9, 13–17]. On the contrary, mouse IM have a heterogeneous developmental origin, arising mainly from circulating and lung monocytes, with only minimal contribution of yolk sac precursors early in fetal life [18].

In contrast to mouse lung macrophages, little is known about the development and homeostasis of human AM and IM. This gap in knowledge is due to the limited availability of appropriate experimental methods and can only be addressed in the context of bone marrow or lung transplantation or with the use of humanized mouse models. Studies in allogeneic bone marrow recipients demonstrated that myeloablation and thus, host AM depletion prior to transplantation induces fast AM replacement with the final AM pool deriving mostly from donor-derived circulating monocytes [19, 20]. Upon lung transplantation, AM are transferred along with the donor lung in the recipients with their longevity impacting donor-specific immune responses and potential long-term graft rejection [21, 22]. Similarly, the origin of human IM is poorly understood, although mounting evidence highlights the contribution of circulating monocytes to human lung macrophage ontogeny in general [23]. Indeed, using a humanized mouse model, Evren et al. demonstrated that CD14^+^CD16^–^ circulating monocytes can extravasate into the lung and give rise to human IM and AM in adults, especially upon lung injury and inflammation [24]. Moreover, the authors showed that the monocytic maturation to IM and AM is characterized by a sequential upregulation of CD206 and CD169, a finding in agreement with human studies reporting that CD206^+^CD169^−^ IM develop earlier than CD206^+^CD169^+^ AM which appear upon lung inflation shortly after birth [24, 25]. More evidence supporting the temporal and tissue state-dependent origin of human AM was recently provided by Evren et al. who characterized the embryonic origin of AM and pinpointed CD116^+^ fetal liver cells as human AM precursors in early life and steady-state conditions [26].

### Dendritic cells professional first responders and immune response initiators

As key antigen-presenting cells (APC), pulmonary dendritic cells (DCs) act promptly upon antigen encounter and trigger adaptive immunity by transporting antigens to lung-draining lymph nodes [7]. In contrast to lung-resident macrophages, insights into prenatal and neonatal development, differentiation and maturation of pulmonary DCs are largely missing. Although DCs are not self-expanding and their tissue maintenance relies on bone marrow-derived replacement, they are considered part of lung-resident immunity given their long tissue persistence and slow replenishment [7].

Three main lung-resident DC subtypes have been identified, namely plasmacytoid DCs (pDCs), monocyte-derived DCs (moDCs), and conventional DCs (cDCs), which can be further subdivided in CD103^+^ cDCs (cDC1s) and CD11b^+^ cDCs (cDC2s) [5, 7, 27]. CD103^+^ cDCs are responsible for sampling and presentation of antigens from the alveolar space to CD8^+^ (and CD4^+^) T cells thereby inducing enhanced effector CD8^+^ T cell generation [28]. Interestingly, in the human lung, the CD103^+^ cDC equivalent subset seems to be the myeloid type 2 DCs (CD11c^+^BDCA-3^+^(CD141) [29, 30]. CD11b^+^ DCs and moDCs express many common markers and thus, can be difficultly distinguished. Nevertheless, the Fc receptors CD64 and/or FcεRIα are commonly used to further distinguish these subtypes [7]. moDCs are recruited to the lung upon inflammation, while their pulmonary existence in steady-state is unclear. Myeloid type 1 DCs (CD11c^+^CD1c^+^) are the human equivalent population to CD11b^+^ mouse DCs [29, 30]. The last main DC subgroup in both mouse and human lungs are pDCs. In mice, pDCs can be identified based on the expression of distinct markers like the plasmacytoid dendritic cell antigen-1 (PDCA-1) [7, 27]. In human lungs, pDCs are characterized as CD11c^−^BDCA-2^+^ [29, 30], while both in mice and humans, pDCs express the antiviral factor bone marrow stromal antigen-2 (BST-2) [31].

### Innate lymphoid cells at the interface between innate and adaptive immunity

Innate lymphoid cells (ILCs) are a group of diverse innate immune cells with a common lymphoid origin that lack antigen-specific receptors and are thus, not implicated into antigen-specific immune responses [32, 33]. Though a small part of lung-resident immunity, ILCs play a pivotal role in mounting and sustaining protective immune responses against invading pathogens while safeguarding tissue homeostasis [34, 35]. If dysregulated, ILCs may also contribute to respiratory disease pathogenesis [36, 37].

The ILC family consists of five main cell subsets with a common lymphoid origin and distinct phenotype and function, namely ILC1s, ILC2s, ILC3, natural killer (NK) cells, and lymphoid tissue inducer (LTi) cells. Based on developmental, phenotypic, and functional similarities, these subsets can be further classified into three groups, with group 1 comprising ILC1s and NK cells, group 2 referring to ILC2s and group 3 consisting of ILC3s and LTi cells [38]. ILCs are considered as innate counterparts of T lymphocytes, with ILC1s, ILC2s, and ILC3s being functionally analogous to CD4^+^ T helper (Th)1, Th2, and Th17 cells, respectively, while NK cells are cytotoxic cells functionally resembling to CD8^+^ T cells [33, 39, 40]. Finally, LTi cells induce secondary lymphoid organogenesis, with their function starting in fetal life [41].

ILCs originate from a common lymphoid progenitor, with their development mainly occurring in the fetal liver and the bone marrow postnatally [42]. Long-lasting maintenance of tissue-resident ILCs is mostly achieved by local self-renewal, but ILC replenishment by bone marrow- or lymphoid organ-derived precursors can also occur [43]. While the lung emergence of ILC1s is poorly understood, ILC2s and ILC3s populate the lung early in postnatal life, with interleukin (IL)-33 production by type II alveolar epithelial cells being critical for ILC2 emergence in the lung [44], and insulin-like growth factor 1 deriving from alveolar fibroblasts promoting ILC3 development, respectively [45]. ILCs reside in all parts of the respiratory tract (Fig. 1). Physiologically, ILC2s are the main ILC population in the mouse respiratory tract, while ILC3s are the predominant ILC subset in humans [46].

**Fig. 3:**
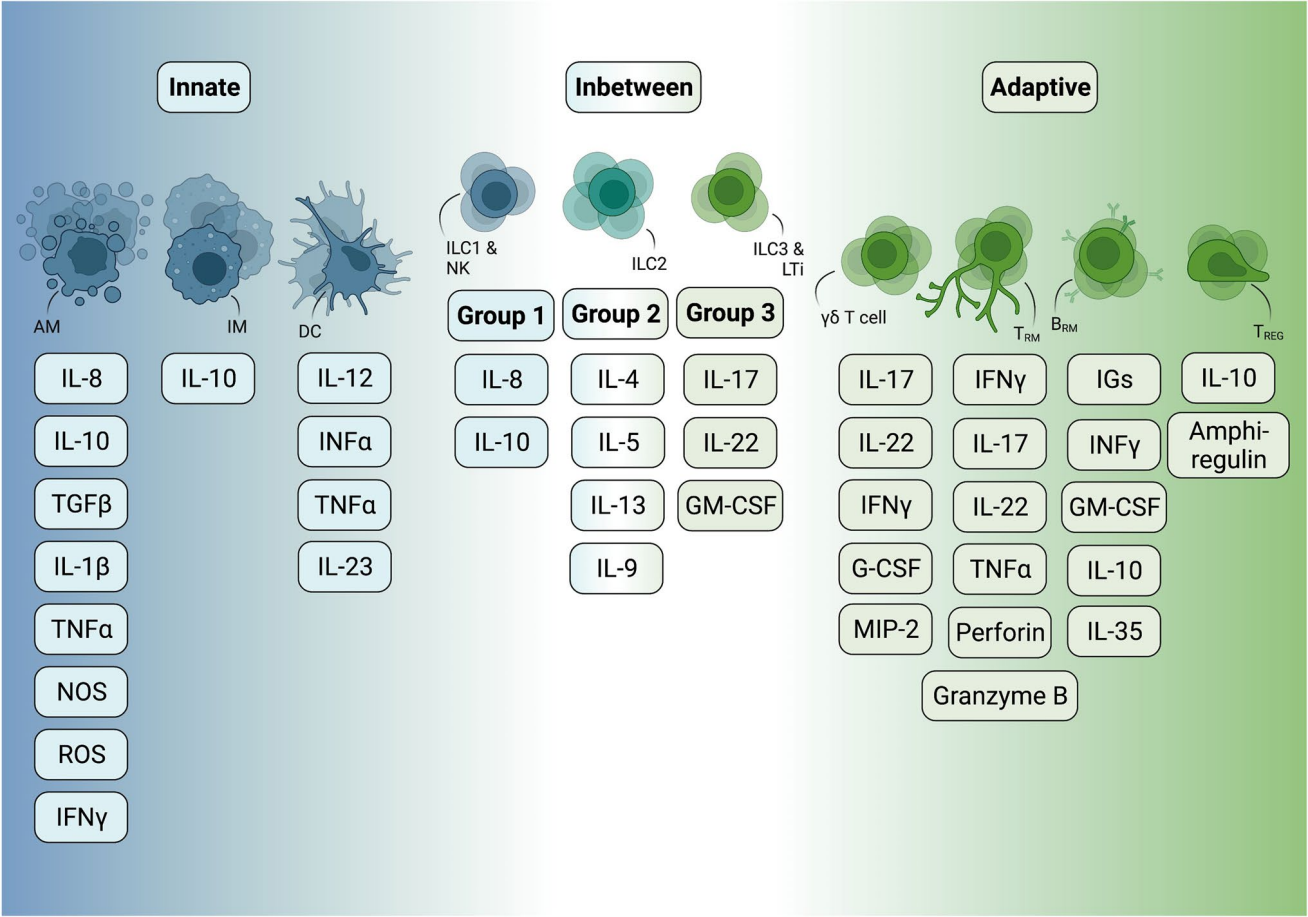
Different subtypes of tissue-resident immune cells and their cytokine production. Created with BioRender.com

Group 1 ILCs comprise NK cells, found in blood circulation and tissues, and ILC1s, which are tissue-resident cells located in several organs, including the lung [43, 47]. Group 1 ILCs are typically implicated in antiviral and antitumor immunity [48–50]. They confer protection mainly through interferon (IFN)-γ secretion in response to IL-12, IL-15, or IL-18, which in turn boosts intracellular pathogen elimination and antigen presentation by other immune cells [49, 50] (Fig. 3). NK cells can additionally secrete perforin and granzyme B, two key mediators of their cytotoxic functions [51]. While expression of tissue residency markers, such as CD103 and CD69, mainly characterizes ILC1s, markers associated with blood recirculation, namely CD62L, sphingosine-1-phosphate receptor (S1PR) and CC-chemokine receptor 7 (CCR7), are mostly expressed by NK cells [33, 52].

As the innate counterpart of Th2 cells, ILC2s are involved in type 2 immunity, due to their ability to produce type 2 cytokines, such as IL-5, IL-4, and IL-13, upon stimulation by epithelial alarmins, including IL-33, IL-25, and thymic stromal lymphoprotein (TSLP) (Fig. 3) [38, 53, 54]. Hence, ILC2s play key roles in asthma and helminth infection [55, 56]. In mice and humans, ILC2 development, maintenance and function depend on the transcription factor GATA3 [57, 58]. Interestingly, ILC2s exhibit tissue-specific phenotypic and functional traits, which are mostly determined perinatally [59, 60]. Schneider et al. reported the existence of fetally, perinatally, and adult-derived ILC2s in adult tissues and identified the perinatal period as a key determinant of tissue-resident ILC2s and their distinct tissue-specific gene expression signature [60]. Of note, only 5–10% of lung-resident ILC2s were of embryonic origin 2 months after birth, indicating that the pool of fetal ILC2s is constantly diluted by postnatally de novo generated ILC2s [60]. Importantly, a tissue-specific de novo generation or local expansion of adult ILC2s in physiological or pathogenic conditions, respectively, was demonstrated, thereby suggesting that the local tissue microenvironment can define the tissue-specific profile of ILC2s in adult life [60]. Apart from their temporal origin, ILC2s exhibit a tissue-dependent responsiveness to IL-33 and IL-25, with IL-33-responding ILC2s residing mainly in the lung and adipose tissue in steady-state conditions and IL-25-responding ILC2s mainly found in the intestine upon helminth infection [44, 59]. ILC2s are the main ILC population in the mouse lung. In steady-state conditions, mouse lung ILC2s express several surface markers, including IL7Ra, CD25, ST2, CD69, CD90, and CD44, while being lineage negative [34, 61]. Despite their similarities, mouse and human ILC2s differ in CD44 and CD161 expression, with mouse cells expressing the first and human ILC2s the second surface marker [33].

Group 3 ILCs, namely ILC3s and LTi cells, depend on the retinoic acid-related orphan receptor-γt (RORγt) for their activation and induce Th17-like immune responses with secretion of IL-17, IL-22, GM-CSF and/or tumor necrosis factor-α (TNFα) (Fig. 3) [38, 62]. ILC3s are characterized by functional and phenotypic heterogeneity. Among others, IL-18 and GATA3 can induce ILC3 maintenance, proliferation, and cytokine production [63, 64]. As key IL-22 producers, ILC3s play an important role in lung homeostasis by sustaining epithelial barrier integrity and function [38, 65].

## Lung tissue-resident adaptive immunity

### Tissue-resident memory T cells specialized sentinels of lung-specific immune memory

The lungs are enriched in both CD4^+^ and CD8^+^ tissue-resident memory T (T_RM_) cells [66, 67], with distinct transcriptional profiles and functional properties [68]. T_RM_ cells are pivotal sentinels of tissue homeostasis, due to their ability to respond rapidly to secondary infections [69] and their role in antitumor local immunosurveillance [70]. However, if dysregulated, T_RM_ cells may also lead to pathogenic immune responses, as seen in the case of allergic asthma [71–74].

Typical T_RM_ cell characteristics include their ability to adhere to peripheral tissues such as the lung and the gut, as well as their lack of homing signals. The phenotypic identification of T_RM_ cells relies on the differential expression of typical surface markers, with CD69 being the most commonly used one for mouse and human T_RM_ cells. CD69 is also a key determinant of T_RM_ cell fate, since it competitively interacts with S1PR thereby inhibiting its expression and impeding sphingosine-1-phosphate (S1P1)-mediated tissue escape [75, 76]. T_RM_ cell tissue retention and inhibition of recirculation are also facilitated by CD44 and CD103 upregulation as well as CD62L and CCR7 downregulation, respectively [77, 78].

Lung T_RM_ cells mainly arise from effector T cells following their DC-mediated activation and subsequent migration from lymphoid tissues into the lung [79, 80]. In the inflamed lung, recruited CD8^+^ effector T cells interact with the local tissue microenvironment, with subsequent differentiation into lung CD8^+^ T_RM_ cells and accumulation at tissue regenerative sites [81, 82]. Lung monocytes and regulatory T cells as well as cytokines secreted by the local tissue microenvironment, such as TGF-β, IL-33, IL-15, TNF, and IFN-γ, play a pivotal role in T_RM_ differentiation and tissue retention [75, 76, 83, 84]. Specifically, TGF-β has been shown to promote important steps for the acquisition of a tissue-resident phenotype, namely CD103 and CD69 expression along with the downregulation of Kruppel-like factor 2 (KLF2) and sphingosine-1-phosphate (S1P1) [76]. During CD8^+^ T_RM_ cell differentiation, T-box transcriptional factors, comprising eomesodermin (Eomes) and T-bet, are also downregulated with minimally sustained T-bet expression required for long-term T_RM_ survival [75]. In contrast to the generation of CD8^+^ T_RM_ cells in other barrier tissues [85], important for the CD8^+^ effector T cell differentiation into lung T_RM_ cells is their prior interaction with cognate antigen, which in turn induces upregulation of CD69, VLA-1, and CD103 [78, 86]. As shown also in other tissues including the liver, skin, kidney, and small intestine [87], a key player in the formation of lung CD8^+^ T_RM_ cells is B-lymphocyte-induced maturation protein 1 (Blimp-1), which shifts the lineage choice of CD8^+^ effector T cell towards T_RM_ and not central memory cells [88].

Despite more extensive investigation of CD8^+^ T_RM_ cell biology, CD4^+^ T_RM_ cells are a more abundant T_RM_ population in the lung and were the first identified and characterized resident memory CD4^+^ T-cell subset [89, 90]. CD4^+^ and CD8^+^ T_RM_ cells share many phenotypic similarities [77] but differ in their generation, surface marker expression and response to cytokines [91]. Although co-expression of CD69 and CD103 is a typical T_RM_ signature and can be used for the identification of lung CD8^+^ T_RM_ cells, lung CD4^+^ T_RM_ cells exhibit high CD69 but only low or no CD103 expression [89, 90]. In contrast to their CD8^+^ counterparts, CD4^+^ T_RM_ generation is affected mainly by IL-2 and IL-15 rather than TGF- β [90, 92].

Lung CD4^+^ and CD8^+^ T_RM_ cells reside in specific tissue sites that support their longevity (Fig. 1). CD8^+^ T_RM_ cells occupy newly constructed niches, also known as repair-associated memory depots (RAMDs), which are associated with tissue regeneration upon injury and are critical for CD8^+^ T_RM_ cell survival [81, 82]. On the other hand, CD4^+^ T_RM_ cells contribute to the formation of inducible bronchus-associated lymphoid tissue (iBALT), which in turn favors their maintenance while providing an immune network that can rapidly respond upon infection [93] (Fig. 1). Nevertheless, lung T_RM_ cells are not as long-lived as in other organs and slowly decline after their generation with constant replacement by circulating effector T cells locally converting into T_RM_ cells [94].

### BRM cells, key features of tissue-specific humoral immunity

**Fig. 4:**
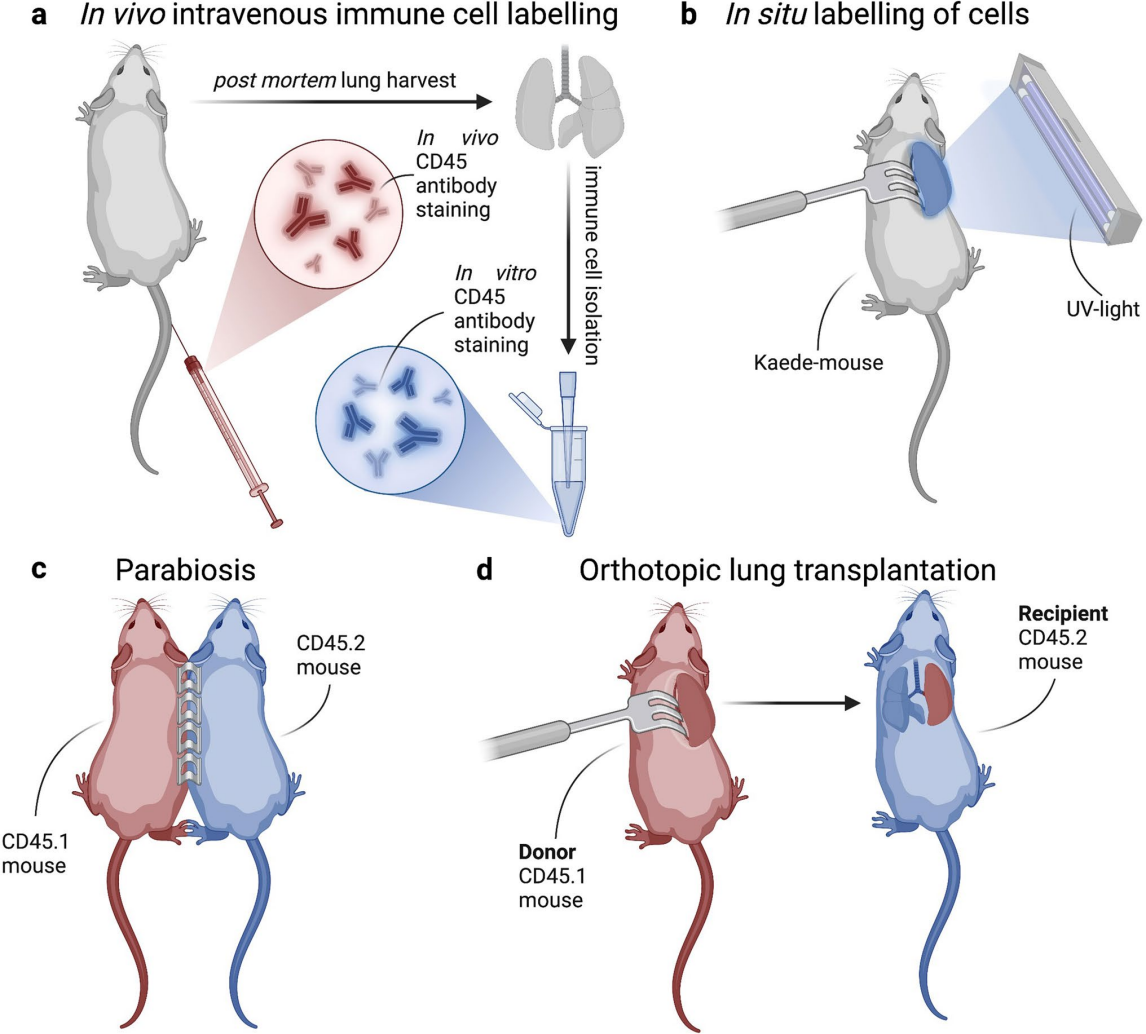
Models to study immune tissue-resident cells in the lung. **a** In vivo intravenous immune cell labelling. **b** In situ labelling of cells through photoconversion. **c** Parabiosis model. **d** Orthotopic lung transplantation. Created with BioRender.com

Tissue-resident B memory (B_RM_) cells are a subgroup of experienced B memory antigen-specific cells, which become unable to recirculate and reside in the lung by altering the expression of their receptors and chemokines [95]. Due to lack of typical B_RM_ markers, identification of lung B_RM_ cells can be challenging and can be mainly achieved through immunohistochemical tissue staining or with the use of intravascular staining and parabiosis animal models (Fig. 4) [3]. In mice, B_RM_ cells share surface markers with B memory cells such as CD38 and CD73 [96], while in humans, they express CD27 [97]. Lung B_RM_ cells express CXCR3, CXCL9, CXCL10, CXCL11, and CCR6 [98], which drive and retain them in the lung parenchyma, where they are located in the iBALT or in the basolateral surface of the respiratory epithelium [99] (Fig. 1). Of note, primary B_RM_ cell generation depends on first antigen encounter [3], which occurs in iBALT and drives B_RM_ precursors to stay and survive in the lung as tissue-resident cells [100]. These cells differ both phenotypically and functionally from B cells of the circulation as well as from those located in the lung-draining lymph nodes [3, 98]. Besides the expression of the above mentioned markers, B_RM_ cells differ from the rest B memory cells by downregulating the homing receptor CD62L and upregulating tissue-resident markers, such as CD69 and CD103 [101]. B_RM_ cells mainly produce antibodies following a secondary infection with their response being faster and much more effective compared to that of other antibody-producing B cells [3].

### γδ-T cells, adaptive and innate-like hybrid facilitators of immune surveillance

Lung-resident γδ-T cells respond rapidly to challenges and orchestrate elicited immune responses thereby contributing to antimicrobial protection, tumor surveillance, and tissue repair [102, 103]. Their distinct nature derives from the combination of conventional adaptive properties with their ability to mount robust innate-like responses [102].

While constituting only 1–5% of blood circulating lymphocytes, γδ-T cells are highly enriched in mucosal and epithelial tissues, such as the skin and the lung, where they account for 8–20% of resident pulmonary lymphocytes [104]. During the perinatal period, Vγ6/Vδ1-expressing γδ-T cells migrate from the thymus to other tissues, including the lung [104, 105], with subsequent tissue-specific differentiation and age-dependent pattern of Vγ gene usage [106]. Specifically, Vγ6^+^ γδ-T cells are the main γδ-T cell population in the lung during the first 8–10 weeks of life, while Vγ4^+^ γδ-T cells become the most abundant γδ-T cell subset after that time point [106]. In the adult mouse lung, γδ-T cells are a heterogeneous population consisting mostly of Vγ4^+^ cells, two smaller Vγ6^+^ and Vγ1^+^ cell subsets, and only sparse Vγ7^+^ cells [107]. This programmed rearrangement of the Vγ gene pattern seems reasonable, considering that γδ-T cells interact with a vast variety of antigens, which depend both on the tissue of residence as well as on the age of the host, thereby conferring targeted conditionally adjusted immunity. Apart from the airway mucosa, lung γδ-T cell can be found in all non-alveolar regions (Fig. 1). Of note, Vγ4^+^ and Vγ1^+^ γδ-T cells are preferentially distributed in parenchymal lung areas [107].

Lung-resident γδ-T cells produce mainly IL-17 [103]. Thus, IL-17-mediated signalling seems to be a key mechanism underlying γδ-T cell contribution to pathogen elimination and pulmonary homeostasis [108], while IL-22 production by γδ-T cells underlies their protective function against lung fibrosis in mice [109]. However, lung γδ-T cells can be also involved in aberrant immune responses and thus disease pathogenesis, as seen in the case of allergic asthma [110].

## Tissue-resident immunity in respiratory diseases

Tissue-resident immunity serves multiple roles in respiratory health and disease. In this section, we discuss the involvement of all above mentioned lung-resident immune cell subtypes in respiratory viral and bacterial infections, asthma, as well as cancer and metastasis (Table 1).

**Table 1:** Summary of the relevance and different functions of lung-resident immunity in respiratory diseases

Disease	Lung-resident subtypes	Functional relevance	References
Respiratory infections	Alveolar macrophages (AM)	Pro-inflammatory cytokine production Triggering of further immune response Enhanced pathogen clearance Anti-inflammatory function Inflammation resolution and tissue repair	[10, 13, 111–116, 139, 155, 156, 183–188]
	Dendritic cells	Antigen presentation Pro-inflammatory cytokine production Triggering of further immune response Enhanced pathogen clearance	[79, 118, 120–123, 140, 192, 193]
	Innate lymphoid cells (ILCs)		
	• *ILC1s*	Immune surveillance at early infection sites IFN-γ production	[49, 50]
	• *Natural Killer (NK) cells*	IFN-γ production Enhanced pathogen clearance Exacerbated influenza pathology in mice	[124–126, 197]
	• *ILC2*	Increased ILC2s linked with severe RSV infection in infants Enhanced airway hyperresponsiveness (AHR), eosinophilia, mucus production in mouse RSV infection Airway epithelial integrity restoration in mouse influenza virus infection	[34, 144–146]
	• *ILC3s*	IL-17 and IL-22 production Tissue repair post infection Enhanced airway epithelial barrier function Protection against secondary bacterial infections	[127, 128, 195, 196, 215]
	Tissue-resident memory T (T_RM_) cells	Heterosubtypic protection against influenza infection IFN-γ and TNF-a production Enhanced pathogen clearance Protection against re-infection	[69, 89, 90, 129–133, 149, 150, 198, 199]
	Tissue-resident memory B (B_RM_) cells	Rapid antibody production upon re-infection	[98, 134, 201]
	γδ-T cells	Pro-inflammatory cytokine production IL-17 and IL-22 production Induction of type 2 immunity Enhanced tissue repair Elimination of infected host cells Inhibition of viral expansion	[135–138, 152, 153, 202, 218]
Asthma	Alveolar macrophages	*Protective:* Enhanced airway inflammation upon AM depletion in mice *Pathogenic:* Pro-inflammatory cytokine production Enhanced type 2 immunity and eosinophilia Impaired phagocytosis and efferocytosis Impaired anti-inflammatory function	[223–228, 232, 233]
	Dendritic cells	*Protective:* Enhanced pulmonary tolerance via Treg cell generation *Pathogenic:* Induction of type 2 immunity Enhanced airway eosinophilia and AHR	[234–239, 241–243]
	ILCs		
	• *ILC1s*	Increased ILC1s in neutrophilic asthma Suppression of ILC2s Resolution of type 2 inflammation	[248, 257]
	• *NK cells*	*Protective:* Enhanced eosinophilic apoptosis Suppression of ILC2s Resolution of type 2 inflammation *Pathogenic:* Induction of type 2 cytokine production	[143, 255, 257, 259]
	• *ILC2*	Increased ILC2s in eosinophilic asthma Enhanced airway eosinophilia and Th2-mediated inflammation Increased AHR and mucus production Disruption of bronchial epithelial barrier integrity	[37, 56, 247–254]
	• *ILC3s*	Increased ILC3s in neutrophilic asthma IL-17 involved in neutrophilic asthma	[261]
	T_RM_ cells	Enhanced airway inflammation by Th2-T_RM_ cells Disease exacerbation upon allergen re-challenge	[71–73, 90, 262]
	B_RM_ cells	Activation upon allergen re-challenge	[263]
	γδ-T cells	*Protective*: IL-17-producing γδ-T cells attenuate AHR and airway inflammation *Pathogenic:* Enhanced airway eosinophilia, IgE production and AHR Treg cell suppression	[110, 264–266]
Lung cancer and metastasis	Alveolar macrophages	Contribution to pre-metastatic niche and enhancement of lung metastasis formation Association with primary lung tumor growth	[275, 277, 278]
	T_RM_ cells	Robust antitumor immunity through enhanced cytotoxicity IFN-γ and granzyme B secretion CD8^+^CD103^+^ T_RM_ cells linked with improved lung cancer prognosis	[70, 279–288]

### Respiratory viral infections

Viruses, including the influenza virus, the respiratory syncytial virus (RSV) and the severe acute respiratory syndrome coronavirus 2 (SARS-CoV-2), are common causes of respiratory infections. Upon encountering the first line of local defense, namely the respiratory epithelium, a further immune response with implication of several circulating and lung-resident immune cell populations is initiated.

#### Influenza virus infection

Invading influenza viruses can target mouse and human lung-resident AM, which are, nevertheless, less susceptible to infection and exhibit lower virus replication and TNF production compared to monocyte-derived macrophages [10, 111]. Of note, AM depletion upon influenza infection leads to increased pulmonary viral load and worsened disease outcome, suggesting that AM enhance pathogen clearance thereby protecting from severe infection [112]. This AM function is mainly due to type I IFN production, which induces pulmonary monocyte recruitment and controls viral replication [113]. Of note, type I IFN production may also underlie the anti-inflammatory behavior of AM, since it alleviates inflammasome activation, reduces IL-1 and enhances IL-10 secretion by monocyte-derived cells and moDCs [114, 115]. However, influenza-driven AM reduction and dysfunction seem to be mediated by IFN-γ signalling thereby increasing susceptibility to bacterial superinfections [116]. Nevertheless, after influenza infection resolution in mice, protection from a secondary *Streptococcus pneumoniae* (*S. pneumoniae*) infection is conferred by newly recruited monocyte-derived AM that enrich the already lung-resident AM pool [13]. In contrast to AM, little is known about the interaction of IM and the influenza virus. A recent study in cynomolgus macaques demonstrated significantly increased IM accumulation in influenza-infected lungs, thereby indicating a potential role of these cells in antiviral defense and immune response orchestration [117].

As critical orchestrators of subsequent immune responses, DCs are also implicated in anti-influenza immunity. Distinct DC subsets exhibit differential susceptibility to influenza infection, mainly depending on the level of MHC II expression. Therefore, highly MHCII-expressing CD103^+^ and CD11b^+^ cDCs can be easily targeted by the virus, while low expressing pDCs are not affected [118]. MHCII molecules are known to serve as viral receptors or co-receptors [119], and this may likely explain the observed differences in DC susceptibility since the entire molecule or at least a region may be recognized by the influenza virus and enhance its binding to DCs. Of note, the infected migratory CD103^+^ cDCs (cDC1s) are the main APCs to lymph node-residing CD4^+^ and CD8^+^ T cells [79, 120]. Interestingly, cDC1 depletion in influenza-infected mice impaired viral clearance and exacerbated disease outcome [120]. Of note, cDC1s also promote CD8^+^ T cell survival in the lungs and development of robust memory response upon influenza infection [121]. However, at later stages of influenza infection, CD11b^+^ cDCs (cDC2) tend to gather in the lung-draining lymph nodes and become the main CD8^+^ T cell-stimulating DC subset [122]. Interestingly, Bosteels et al. recently demonstrated that, upon a respiratory viral infection, pulmonary cDC2s acquire a hybrid phenotype in a type I IFN-dependent manner, sharing expression of the Fc receptor CD64 and of interferon regulatory factor 8 with monocytes and cDC1s, respectively, and exhibit then an enhanced ability to prime CD4 and CD8 immunity [123].

Group 1 ILCs, namely ILC1s and NK cells, contribute to antiviral immunity, mainly through IFN-γ secretion [49, 50]. Of note, NK cells seem to play an ambiguous role upon influenza infection. Although several studies report higher pulmonary virus titers in influenza-infected NK cell-deficient mice [124, 125], a pathogenic impact of NK cells, exacerbating influenza-related pathology, has also been described [126]. In contrast, ILC1s serve a key role in immunosurveillance at sites of early viral infection and protect by secreting IFN-γ in response to cDC1-mediated signaling [50]. Additionally, in H1N1-influenza-infected mice, ILC2s enhance airway epithelial restoration by boosting epithelial cell proliferation and airway remodeling via amphiregulin and IL-13 production [34]. Although little is known about the role of ILC3s in viral respiratory infections, the implication of IL-22, a key ILC3 cytokine, in influenza infection has been thoroughly investigated. IL-22 contributes to lung epithelial repair following infection [127]. Additionally, IL-22 and thus IL-22-producing ILC3s confer protection against secondary bacterial lung infections [128].

Mouse and human studies identify virus-specific T_RM_ cells in influenza-infected lungs [89, 90, 129], which fight acute infection through IFN-γ and TNF-a production [130] and support long-term protective immunity in situ [131]. Pulmonary influenza-specific CD8^+^ T_RM_ cells confer also heterosubtypic protection against infection due to the presence of common or similar epitopes [69, 129, 132]. Indeed, CD8^+^ T_RM_ cells with the ability to confer cross-protection against influenza A, B, and C viruses have been found in human lungs following influenza infection [129]. Interestingly, the development of lung-resident CD8^+^ T_RM_ and B_RM_ cells upon influenza virus infection is supported by a newly identified population of follicular tissue-resident CD4^+^ T helper (T_RH_) cells in an IL-21-dependent manner [133]. Apart from T_RM_ cells, long-lived B_RM_ cells are also found in influenza-infected mouse and human lungs [3, 98, 134]. Importantly, influenza-specific B_RM_ cells reside in iBALT and exhibit distinct transcriptional and phenotypic traits, including increased CXCR3, CCR6 and CD69 expression, which distinguish them from populations in lung-draining lymph nodes, spleen, or blood [98]. Upon viral re-exposure, pulmonary B_RM_ cells preferentially migrate into infected sites, where they differentiate into plasma cells with high antibody production [134].

Lung-resident γδ-T cells are also involved in antiviral immunity. Upon neonatal influenza infection in mice, epithelial cell-derived IL-33 triggered the accumulation of IL-17A-producing γδ-T cells in the infected lungs. In turn, these cells induced type 2 immunity, ILC2 and regulatory T (Treg) cell recruitment and, finally, enhanced amphiregulin release and tissue repair [135]. In humans, the main γδ-T response against influenza virus infection is exerted by IFN-γ-producing Vγ9Vδ2-T cells [136]. Indeed, activated human Vγ9Vδ2-T cells could kill influenza-infected human alveolar epithelial cells and impede viral replication in vitro [137], while they could also attenuate disease severity in immunodeficient, infected with human influenza virus strains, humanized mice [138].

#### RSV infection

Similar to the influenza virus infection, tissue-resident immunity is critically involved in immune responses elicited upon a RSV infection. As sentinels of respiratory barrier immunity, both AM and pDCs enhance viral clearance and thus protect from severe RSV infection [139, 140]. Indeed, AM or pDC depletion upon RSV infection led to increased lung viral load and aggravated disease pathology [139, 140]. The contribution of AM to viral clearance is mainly due to type I IFN production [113, 141]. In fact, AM seem to produce more type I IFN than pDCs, and thus play a superior role in antiviral immunity [113]. Interestingly, AM not only initiate immune response upon RSV encounter, but are also able to limit viral replication regardless of type I IFN [142].

Similar protective roles have been described for group 1 ILCs upon RSV infection [143, 144]. On the contrary, increased ILC2s have been associated with severe RSV bronchiolitis in infants [145], and correlated with high TSLP and IL-33 levels [145]. Upon mouse RSV infection, early TSLP-mediated induction of IL-13-secreting ILC2s led to airway hyperresponsiveness (AHR) and increased mucus production [144]. Similarly, Wu et al. recently demonstrated that RSV-triggered IL-33-activated ILC2s boost AHR and airway eosinophilia through IL-13 production [146]. Interestingly, increased ILC2 numbers persist in the mouse lung for several weeks post neonatal RSV infection [146, 147], and thus may account for the link between early-life RSV infection and long-term respiratory pathologies [148].

On the other hand, a protective role of virus-specific CD8^+^ T_RM_ cells has been described upon RSV infection in both mice and humans [149, 150]. In RSV-immune mice, T_RM_ cells amplified virus clearance thereby protecting against re-infection [150]. RSV infection in humans led to accumulation of virus-specific CD8^+^ T_RM_ cells in the lungs during convalescence, while increased RSV-specific T_RM_ cells in the airways before infection were associated with reduced virus titers and disease severity [149]. Virus-specific CD4^+^ T_RM_ cell accumulation in the airways has also been reported upon experimental human RSV infection [151].

Upon RSV-immunization and subsequent infection, increased Vγ4^+^ γδ-T cells were found in mouse lungs and could produce several pro-inflammatory cytokines, including IFN-γ, TNF, IL-4, and IL-5, upon ex vivo stimulation [152]. Of note, depletion of these cells attenuated lung immunopathology and disease severity and minimally enhanced viral proliferation without affecting viral clearance [152]. Additionally, RSV-infected mouse neonates failed to mount robust IL-17A-mediated responses, in contrast to adult mice, in which γδ-T cells were the main IL-17A sources. Importantly, IL-17 suppression in adult mice exacerbated RSV-induced lung inflammation, whereas IL-17 supplementation in neonates had the opposite effect. Thus, increased RSV disease severity in infancy may be associated with an early-life deficiency or dysfunction of lung IL-17A-producing γδ-T cells [153].

#### SARS-CoV-2 infection

Direct SARS-CoV-2 infection of human AM has also been reported [154], with subsequent AM dysfunction, exacerbated pro-inflammatory responses and eventually generalized pulmonary damage [154]. Upon severe coronavirus disease (COVID-19), AM expand into the alveoli along with neutrophils and lymphocytes, and seem to sustain an M1 polarization thereby contributing to SARS-CoV-2-associated “cytokine storm” and acute respiratory distress syndrome [154, 155]. Recently, Grant et al. identified a positive feedback loop between SARS-CoV-2 infected AM and T cells whose continuous interaction perpetuates a spatially restricted alveolitis [156]. Of note, in vitro SARS-CoV-2 infection of monocyte-derived macrophages and dendritic cells led to production of cytokines, such as IFN-α, IFN-β, TNF, IL-1β, IL-6, IL-10, and CXCL10 and also resulted in type I IFN-mediated cell death, despite its abortive character and the fact that the virus failed to replicate efficiently in these cells [157]. However, SARS-CoV-2-induced IFN production was weaker compared to other respiratory viruses [158], a finding that may be attributed to newly identified SARS-CoV-2-encoded genes limiting IFN production [159] as well as a poor ability of AM to sense the invading virus and mount a robust IFN response [160].

Upon SARS-CoV-2 encounter, DCs seem to be unexpectedly affected. Interestingly, a lower DC number, impaired antigen-presenting function and decreased type I IFN production by DCs have been described in patients with severe COVID-19 [161–163]. Among cDC1s, cDC2s, and pDCs, only cDC2s accumulate in SARS-CoV-2-infected human lungs [162], while pDC apoptosis is increased [162, 164]. The preferential accumulation of cDC2s in SARS-CoV-2-infected lungs may be explained by the fact that cDC2s can enhance the function of CD4^+^ T cells and trigger follicular helper T cells, which both contribute to effective antiviral humoral immune responses, as shown in the case of human immunodeficiency virus-1 infection [165]. Of note, upon acute SARS-CoV-2 infection, DCs failed to induce robust T cell responses, thus suggesting an impaired antigen-presenting capacity [163, 164]. A similar DC dysfunction could be seen in SARS-CoV-infected rhesus macaques. In that case, the low rate of viral replication in all infected APCs and the consequently low expression of viral proteins impaired viral sensing from Toll-like receptors (TLRs) thereby facilitating viral escape from mucosal innate immunity and subsequent systemic dissemination using APCs, including DCs, as vehicles [166]. Additionally, SARS-CoV-2 preferentially inhibits pDCs from producing type I IFN and can thus escape immune recognition [163, 167].

Our understanding of ILC implication in COVID-19 is limited to changes observed in peripheral blood and thus, insights into lung-resident ILCs are sparse. Higher expression of activation and homing markers in ILCs from infected individuals was associated with severe infection [168]. This finding together with decreased NK, ILC1 and ILC2 numbers in the blood of COVID-19 patients [169] suggest that ILCs migrate and persist in the lung upon severe infection.

SARS-CoV-2 infection also induces development of lung-resident adaptive immune memory [170, 171]. Human studies report a long-term persistence of virus-specific lung T_RM_ cells up to 10 months after primary infection [170]. Importantly, cross-reactive immunity has also been described in the case of SARS-CoV-2, as, due to its homology with the human coronaviruses OC43 and 229E, cross-reactive CD4^+^ and CD8^+^ T cells have been detected in the absence of a prior SARS-CoV-2 infection [172–174]. Although both in the case of influenza or SARS-CoV-2 infection, virus-specific lung T_RM_ cells seem to be generated and cross-react with heterologous virus strains, whether these cells confer cross-protection against other antigens remains unclear. Of note, pre-existing immunity to influenza, either due to prior infection or vaccination, seems indeed to impact SARS-CoV-2-specific T-cell immune responses, since it has been associated with enhanced SARS-CoV-2-specific CD4^+^ T cell immunity [175] and reduced rate of COVID-19 [176]. Such cross-immunity can be attributed to the structural similarities of influenza virus and SARS-CoV-2 [177] and thus, lung T_RM_ cells may be key players in this context, a hypothesis warranting further investigation. However, little is known about the ability of SARS-CoV-2-specific lung T_RM_ cells to sufficiently protect against re-infection. Of note, a sublethal mouse SARS-CoV-2 infection induced pulmonary resident CD4^+^ and CD8^+^ T effector cells expressing T_RM_-related markers such as CD69 and CD103, but, when adoptively transferred to a naïve host, these cells failed to independently protect against a lethal SARS-CoV-2 infection [178]. This finding is in contrast to other studies showing that pulmonary resident T cells are essential for vaccine-induced protection against coronaviruses [179]. Taken together, these observations may indicate that, unlike a SARS-CoV-2 infection, a vaccination may induce the generation of lung-resident T cells with enhanced protective capacity against infection. Another explanation for the inability of transferred pre-trained lung-resident T cells to independently protect against infection may be that immune responses commonly rely on well-orchestrated cellular events with immune cells acting as a network. Thus, an independent protective function of one immune cell population may not be sufficient to confer immunity in this case. Interestingly, a recent study by Zhao et al. uncovered a pathogenic role for tissue-resident memory-like Th17 (T_RM_17) cells as potential drivers of aberrant inflammation in severe COVID-19 pneumonia [180]. Specifically, COVID-19 severity and inflammation-induced lung injury were associated with the interplay among T_RM_17 cells, macrophages and CD8^+^ T cells in the infected lungs [180]. Of note, patients with severe disease exhibited increased serum IL-17A and GM-CSF levels [180]. However, a potential protective function of T_RM_17 cells at an early infection stage or in asymptomatic SARS-CoV-2 infected individuals could not be ruled out. Therefore, further studies are required to fully decipher the emergence and origin as well as the role of these cells throughout the infection course.

Studies focusing on γδ-T cell responses upon SARS-CoV-2 infection deal mainly with circulating γδ-T cells and largely neglect the lung-resident subtypes. Of note, decreased circulating γδ-T cells are found in the blood of patients with severe COVID-19 and related general lymphopenia [181]. Additionally, recovery from COVID-19 has been associated with a shift of γδ-T cells towards an effector-like phenotype with enhanced tissue infiltration capacity [182]. Taken together, these findings might indeed suggest a recruitment and retention of γδ-T cells in the infected lung [158, 183].

### Respiratory bacterial infections

Similar to respiratory viral infections, lung-resident immunity is essentially involved in protection against invading bacteria, such as *S. pneumoniae* and *Mycobacterium tuberculosis (M. tuberculosis)*.

#### S. pneumoniae infection

AM are among the first responders to invading bacteria. Upon *S. pneumoniae* infection, TLRs of AMs can synergistically prevent pneumococci from escaping immune recognition [184]. Following phagocytosis, pathogen elimination can be achieved through AM apoptosis [185]. Nevertheless, the elimination of phagocytized pathogens, such *as S. pneumoniae,* most commonly relies on the acidity of the phagosome and AM-produced reactive oxygen and nitrogen species [186]. Gut microbiota-derived acetate was found to boost AM bactericidal activity by inducing production of IL-1β and in turn nitric oxide by AM [187]. AM are also critically involved in inflammation resolution and tissue repair. After infection control, reprogramming of AM towards the M2 activation state results in the secretion of anti-inflammatory cytokines, such as IL-10 and IL-1ra thereby controlling inflammation and boosting tissue repair [188]. Additionally, through AM-mediated efferocytosis, dead cells and their intracellular inflammatory residues are also removed [183, 188]. Regarding IM, several subtypes with distinct transcriptional profiles can be found during a bacterial infection [189] and they all express MHCII, a trait likely indicating an antigen presenting role [190]. Additionally, IM population is known to expand significantly after exposure to bacterial unmethylated CpG DNA and can subsequently prevent asthma development through IL-10 production [191]. Unlike AM, insights into the role of lung-resident IM in respiratory bacterial infections remain sparse and thus, further studies focusing on this topic are needed.

Although the role of DCs in antiviral immunity is well-described, their response to bacterial lung infections is less understood. As first-line defenders, DCs exert their antigen presenting activity also upon bacterial infections [192]. On the other hand, DC deficiency in *S. pneumoniae*-infected mice was associated with reduced systemic bacterial spread and thus, lower systemic inflammation, indicating the ability of pneumococci to exploit DC-mediated proteolysis in order to spread outside the lung [193]. Interestingly, DC depletion was not accompanied by an increased recruitment of another immune cell type [193], and thus, one can only assume that other antigen presenting cells, such as AM, monocytes and epithelial cells undertake the role of DCs in this context [194].

ILCs play also an important role in host responses to bacterial lung infections, with ILC3s being the most important ILC population in this context [195]. Upon infection, pro-inflammatory factors such as IL-1β and IL-23, trigger ILC3s to secrete IL-17 and IL-22 [195], which subsequently enhance airway epithelial barrier function and promote immune responses against *S. pneumoniae* [196]. Apart from ILC3s, NK cells contribute also to bacterial clearance, likely due to their ability to produce IL-22 and IL-15 [197]. On the other hand, ILC2s seem to indirectly impede immune response to *S. pneumoniae* by skewing AM phenotype and function towards an M2 activation state thereby favoring immune quiescence in homeostasis [44].

T_RM_ and B_RM_ cells are also integral parts of immune defense against bacterial pneumonia. Bacterial respiratory infections lead to IL-12/IL-18-mediated bystander activation of virus-specific lung CD8^+^ T_RM_ cells, which in turn secrete IFN-γ and attract neutrophils into the lung thereby reducing disease severity [198]. Importantly, neutrophil recruitment in *S. pneumoniae*-infected lungs can also be boosted by CD4^+^ T_RM_ cells, which, in response to pneumococcal antigens, produce IL-17 and reprogram lung epithelial transcriptome to accelerate antimicrobial responses [199]. Interestingly, treatment with antibiotics such as clarithromycin was associated with a reduced lung CD4^+^ T_RM_ cell number and thus impaired host response to a *S. pneumoniae* re-infection. Such an impairment of tissue-resident immune memory cells may be a mechanism, underlying the long-term ability of extensive antibiotic use to dysregulate immune responses and weaken the host’s defense against a re-infection [200]. B_RM_ cells are also elicited in human and mouse lungs after pneumococcal infection and their depletion from experienced mouse lungs prior to a re-infection has been associated with increased disease severity [201].

As IL-17 sources, lung resident γδ-T cells are essentially involved in host immune defense against bacterial infections. Additionally, γδ-T cells contribute to the resolution of pneumococcal inflammation, since they can expand and drastically reduce the number of lung DCs and AM following *S. pneumoniae* clearance [202].

#### M. tuberculosis infection

As in the case of pneumococcal infection, AM are essentially involved in the elimination of *M. tuberculosis* upon infection*.* The central TLR adapter protein myeloid differentiation factor 88 (MyD88) and the macrophage receptor with collagenous structure (MARCO) are essential for elimination of both *S. pneumoniae* and *M. tuberculosis* [203–205], by, among others, mediating AM interaction with another integral sensor of tissue-specific defense, namely the lung epithelium [204]. Invading mycobacteria are easily recognized and phagocytosed by AM, which subsequently secrete pro-inflammatory cytokines and exert bactericidal effects by highly expressing inducible nitric oxide synthase and antimicrobial agents [206]. Despite their –largely- protective role in bacterial respiratory infections, upon a *M. tuberculosis* infection, IL-1-mediated interplay between infected AM and non-hematopoietic cells facilitates the migration of the former from the alveolar space to the lung interstitium thereby favoring tuberculosis progression [207]. AM ability to support *M. tuberculosis* intrapulmonary dissemination can be explained by the fact that *M. tuberculosis* bacilli can survive in the phagosomes of AM, and thus AM depletion in *M. tuberculosis*-infected mice enhances bacterial clearance and ameliorates disease outcome [208].

DCs are also critically involved in host defense against *M. tuberculosis*. Bacillus phagocytosis and induced cytokine production by DCs control the infection and granuloma formation [209]. Infected DCs migrate to draining lymph nodes and trigger adaptive immune responses [210]. Mycobacterium-specific Th1 responses are orchestrated by CD11b^+^ cDCs, while at the same time being suppressed by CD103^+^ cDCs via IL-10 production [211]. Additionally, moDCs highly express pro-inflammatory cytokines to support mycobacterium elimination and enhance Th2 and Th17 immune responses [212]. However, little is known about the role of pDCs in tuberculosis, although their number increases in lung-draining lymph nodes upon mycobacterial infection [213].

Recent evidence has uncovered a critical implication of ILCs in antimycobacterial immunity. Studies in mice demonstrate a protective role of lung-resident IFN-γ-producing NK cells in *M. tuberculosis* infection, which can bind on the pathogen and exert cytotoxic effects [214]. Additionally, early innate immune responses to *M. tuberculosis* are further facilitated by ILC3s via IL-17 and IL-22 production [214, 215].

The presence of antigen-specific IL-17-producing CD4^+^ and CD8^+^T_RM_ cells in *M. tuberculosis*-infected human lungs has also been recently reported [216], an intriguing finding raising discussions about the efficiency of tissue vaccines against tuberculosis, in an effort to boost T_RM_ cell presence in the passively immunized lung [217]. Finally, through IL-17 production, lung γδ-T cells also play an important role in host immune response against *M. tuberculosis,* including granuloma formation [218, 219].

### Asthma

Asthma is one of the most widespread respiratory conditions worldwide. Its pathogenesis is driven by chronic airway inflammation, AHR and remodeling which overall result in episodic manifestation of respiratory symptoms including wheezing, coughing, and dyspnea. Due to improved understanding of the disease, asthma is now considered as a heterogeneous entity consisting of distinct disease endotypes and phenotypes [220]. Asthma endotypes refer to underlying inflammatory pathways that result in the clinical features of the disease, namely the asthmatic phenotypes [220, 221]. The most well-recognized and defined endotypes of asthma are the type 2-high and type 2-low endotypes, characterized by eosinophilic or neutrophilic/paucigranulocytic airway inflammation, respectively [220–222]. Patients with eosinophilic or neutrophilic asthma share similar clinical symptomatology but different endotypes need to be taken into account for optimal selection of treatment strategies [221, 222]. Although different molecular and immune cell pathways predominate distinct disease endotypes, lung-resident immunity is an essential part of all asthma-related immune responses.

AM seem to play a rather dichotomous role in asthma pathogenesis. Although AM depletion exacerbated allergic airway inflammation (AAI) in mice [223], AM-produced mediators, including CCL17, CCL8, and CCL24, enhanced type 2 immunity and eosinophilia in asthmatic patients and mice [224]. Additionally, AM from patients with asthma secrete more pro-inflammatory mediators, such as TNF-a, IL-1β, IL-6, IL-8, and IL-17, which promote AAI [225]. In asthmatic patients, the dysfunctional AM phagocytotic and efferocytotic capacity perpetuate inflammation and tissue remodelling and trigger disease exacerbations [226–228]. Of note, an impaired phagocytic capacity is not a general feature of asthma as it correlates with disease severity and depends on the type of product to be phagocytosed [229]. Additionally, AM from patients with severe—but not mild- asthma exhibit a defective efferocytotic capacity, which in turn is associated with an impaired anti-inflammatory function [228, 230]. On the contrary, apoptotic cell efferocytosis by monocyte-derived macrophages from asthmatic patients remains intact [231], a finding that may be attributed to the different cellular origin of lung-resident macrophages [10]. Similarly, lung-resident macrophages exhibit an impaired anti-inflammatory behavior in asthma. Reduced IL-10^+^ macrophages were observed in asthmatic patients [232], while an increase of IL-10^+^ IM could dampen airway inflammation or prevent neutrophilic asthma in mice [233]. However, the exact mechanisms underlying macrophage dysfunction and its impact on asthma manifestation remain elusive and thus, further studies in this field are warranted.

Similar to AM, DCs also contribute to asthma-related immunity. Upon allergen encounter, DCs trigger naïve Th cell differentiation and thus Th2 and ILC2 accumulation with type 2 cytokine production [234]. Both lung-resident cDC1s and cDC2s are able to elicit a strong Th2 response upon house dust mite (HDM) exposure [235–237], while CD103^+^ cDC1-deficient mice exhibit attenuated AHR and eosinophilia upon ovalbumin-induced asthma [238]. Of note, pDCs can be divided into distinct subpopulations based on the expression of the surface markers CD8α or CD8β, which were shown to either prevent or enhance asthma manifestation in mice [239]. Interestingly, expression of these markers can be observed after TLR agonist-mediated stimulation and has been linked with distinct cytokine expression profiles and a tolerogenic DC phenotype [239, 240]. Importantly, DCs can not only trigger and sustain but also control and limit allergic Th2 immune responses [241, 242]. For example, CD103^+^ DCs seem indeed to play an important role in pulmonary tolerance, since they are able to induce Treg cell generation in ovalbumin-challenged mice [241, 242], while their absence has been linked with exacerbated AAI and eosinophilia in both ovalbumin- and HDM-induced asthma [241–243]. Interestingly, regular administration of *Helicobacter pylori* extract to ovalbumin-treated mice attenuated AAI, eosinophilia and AHR, with the success of the treatment relying mainly on CD103^+^ DCs and their intrinsic IL-10 production [244]. Treg cell generation can also be induced by pDCs, which seem to mostly attenuate AAI and promote pulmonary tolerance to harmless inhaled antigens [245, 246].

As well-known type 2 cytokine producers, ILC2s are highly involved in asthma pathogenesis [56]. Indeed, increased blood circulating and lung-resident ILC2s can be found in asthmatic patients [56, 247]. Interestingly, this increase applies mainly to eosinophilic asthma, whereas neutrophilic asthma has been associated with elevated ILC1s and ILC3s [248]. ILC2s boost airway eosinophilia via IL-5 production [249] and enhance AHR, goblet cell hyperplasia and Th2-mediated AAI through IL-13 secretion [37, 250]. Additionally, increased bronchial epithelial barrier permeability and disrupted tight junction integrity have been associated with ILC2s and IL-13 production [251]. A recent study identified the neuropeptide neuromedin U as a powerful effector of AAI through ILC2 activation [252]. On the other hand, several mediators, including IFN-γ, IL-1β, and Nrf2, have been found to tightly control ILC2-mediated immunity thereby preventing or suppressing AAI in mice [250, 253, 254]. Although little is known about the role of ILC1s in asthma, NK cells can be both protective and pathogenic in this context. On one hand, NK cells seem to restrict AAI by promoting eosinophilic apoptosis and impeding viral-induced allergic immune responses [143, 255]. Of note, severe asthma has been associated with decreased lipoxin A4 and thus, impaired ability of NK cells to trigger eosinophil apoptosis [256]. In mice, IFN-γ-producing NK cells and ILC1s can suppress ILC2 expansion and activation thereby contributing to type 2 inflammation resolution [257]. Consistently, NK cell depletion during the early phase of papain-induced lung inflammation in mice led to ILC2 expansion, increased type 2 cytokine production and thus, aggravated asthma manifestation [258]. On the other hand, NK cells can also promote type 2 cytokine production and thus, trigger AAI [259]. Overall, NK cells are critically involved in the phase of sensitization upon allergen encounter, facilitate the balance between Th1 and Th2 inflammation and finally contribute to the resolution of allergic inflammation. The impact of NK cells on these steps depends largely on their activation status and subtype. Of note, the result of NK activation can be influenced by the type of environmental stimulus, the cytokine and inflammatory milieu, the interaction with other parenchymal and immune cells, the differentiation status of the cell and the developmental phase of the host individual [260]. Since immune cells, and in this case NK cells, exert their functions within an interacting network, a modulation of this cross-talk in favor of NK activation or inhibition may be a promising immunotherapeutic approach. A better understanding of the implication of NK cells in allergic asthma including the crucial elements that govern their dichotomous mechanisms of action is thus required. Finally, although insights into the role of group 3 ILCs in asthma are sparse, IL-17-producing ILC3s may be involved, since IL-17 has been associated with neutrophilic asthma in both humans and mice [261].

T_RM_ and B_RM_ cells are also implicated in the pathogenesis of asthma [73, 262]. Specifically, Th2-T_RM_ cells secrete cytokines that promote and sustain airway eosinophilia [72] [262]. Upon HDM exposure, IL-2 signalling enables the generation, migration and retention of allergen-specific Th2-T_RM_ cells in the lungs, where they drive AAI [72]. Of note, these long-lived pathogenic cells remain in the mouse lung for its entire lifetime as safekeepers of allergic memory [71]. Therefore, allergen re-challenge induces rapid Th2-T_RM_ cell proliferation and type 2 cytokine secretion with subsequent exacerbated clinical features of asthma [73, 74]. B_RM_ cells could also be identified in the lungs of HDM-sensitized and challenged mice and were implicated in the allergic response, while HDM re-challenge resulted in their activation [263].

Lung-resident γδ-T cells seem to play a dichotomous role in allergen-induced Th2 immunity. In ovalbumin-induced asthma, γδ-T cell-deficient mice had reduced AHR, airway eosinophilia, peribronchial lymphocytic infiltration as well as lower serum IgE and lung IL-5 levels compared to wild-type mice [264, 265]. Besides promoting airway eosinophilia and regulating IgE production, Vγ1^+^ γδ-T cells boost AHR by suppressing IL-10-producing Treg cells in the lung of ovalbumin-treated mice [266]. However, IL-17-producing γδ-T cells have been identified as key regulators of pulmonary allergic responses, as they could ameliorate AHR thereby enhancing resolution of eosinophilic and Th2-mediated AAI [110, 267]. Indeed, activation of Th17-like γδ-T cells has been associated with lower AHR [268], attenuated airway eosinophilia as well as increased neutrophil airway recruitment [268] and higher AM number [110].

## Tissue-resident immunity in lung cancer and metastasis

Lung cancer is the most frequently occurring type of cancer and the leading cause of cancer-related death in men worldwide, while it has the third highest incidence and second highest cancer-related mortality in women [269]. Apart from primary tumor growth, the lungs are frequently targeted by metastatic tumor cells originating from primary tumors located at other parts of the lung itself or distant sites, such as the breast, colon or the skin [270]. Similar to lung cancer, lung metastasis is a major health burden worldwide and a common cause of cancer-related death [271]. Although the pathogenesis of lung cancer and metastasis has not been fully elucidated yet, mounting evidence identifies the lung microenvironment and the crosstalk between cancer and immune cells as key players in this process [272].

Tissue-resident immune cells can play a critical role in tumor surveillance and immunity. Despite the multifaceted nature of lung-resident immunity, most studies focus on the role of T_RM_ or –to a smaller extent- of tissue-resident macrophages in lung tumorigenesis and metastasis formation, while insights into the potential involvement of other resident immune cell subsets are largely missing.

During tumor or metastasis formation, lung-resident macrophages are among the first cells to interact with tumor cells and may thus play a critical role in this process. Th2-driven inflammation can be typically found at tumor sites and tumor microenvironment (TME) [273], and evidence demonstrates that IL-4 boosts tissue-resident macrophage proliferation [274]. In the mouse lung, monocyte-derived macrophages were shown to induce metastasis, while tissue-resident macrophages were associated with primary tumor growth [275]. Additionally, tumor-associated macrophages (TAMs) in mouse lungs were shown to be tissue-resident IM and CCR2-dependent recruited macrophages, with the former mainly promoting tumor growth in—among others- an IL-9-dependent manner [276], and the latter facilitating tumor cell dissemination [275]. In accordance to these findings, intratracheal L-Clodronate-mediated AM depletion did not affect lung metastasis of mammary carcinoma-derived Met-1 cells [277]. However, a role of AM in metastasis cannot be ruled out, since in a mouse model of breast cancer, AM were found to enhance lung metastasis by impeding antitumor T cell activity in the lung and thus, could be identified as an important resident of the pre-metastatic niche and a potent target of future cancer immunotherapies [278].

Recent findings suggested that tumor-associated lymphocytes in non-small cell lung cancer (NSCLC) exhibit T_RM_ cell function [279]. In TME, CD8^+^ T_RM_ cells comprise a homogeneous CD103^+^ CD49^+^CD69^+^ population characterized by T-bet, porylated (p)STAT-3, and Aiolos transcription factor expression, while a small subset produces IFN-γ and IL-17 [279].

In human NSCLC, cytotoxic CD8^+^ T cells with high CD103 expression exhibit increased cytotoxicity, are highly proliferative, and can thus contribute to robust antitumor immunity [280]. In early-stage NSCLC, effector memory T cells face tumor-related antigens and transform into CD103^+^ T_RM_ cells with antitumor activity [281]. Of note, the function of CD8^+^ tumor-infiltrating lymphocytes (TIL) in this stage seems to be determined by a balance between an antitumor CD103^+^ T_RM_ program and an exhaustion program driven by Eomes expression. During tumor growth, the exhaustion program may prevail, thus reducing TIL function [281].

Furthermore, transcription factors may be also essentially involved in this process. A gradual reduction of TILs as well as progressively reducing nuclear factor of activated T cells (NFATc1) expression in cancer cells have been described in patients with advanced-stage NSCLC [282]. Interestingly, enhanced tumor growth together with decreased effector memory and CD103^+^ T_RM_ cells were observed in tumor-bearing lungs of mice with T-cell-specific NFATc1 inactivation, thus highlighting the role of this transcription factor in cytotoxic T-cell immunity and T_RM_ cell tissue retention [282, 283]. Additionally, CXCR6 surface expression on mouse and human lung T_RM_ cells upon tumor antigen encounter facilitates their migration and maintenance in lung TME [284]. Moreover, CD103^+^ T_RM_ cell-derived granzyme B and IFN-γ in humans control tumor formation and metastasis through fibronectin secretion, facilitate the priming of newly generated tumor-specific T cells, and boost immune cell recruitment in the tumor [285]. Interestingly, enhanced proliferation of CD103^+^ T cells in tumors with high CD8^+^ T cell numbers was associated with a prolonged survival, a finding likely identifying CD8^+^CD103^+^ T_RM_ cell infiltration as a positive prognostic factor [70, 286].

Although several studies report an association of CD8^+^ T_RM_ cells in human NSCLC with a good disease prognosis [283], little is known about the role of CD4^+^ T_RM_-like TILs in this context. CD4^+^ T_RM_ cells are a phenotypically and functional heterogeneous population and thus, multiple, even contradictory, functions in TME are to be expected. Of note, CD8^+^ T cell cytotoxicity largely depends on CD4^+^ T_RM_ cells, which can also hinder tumor growth via IFN-γ production or by tumor cell elimination [287].

Regarding the implication of T_RM_ cells in lung metastasis, Christian et al. recently demonstrated that T_RM_ cells develop in the tumor, the contralateral mammary mucosa, and the pre-metastatic lung. In a functional level, CXCR6 is critically involved in T_RM_ retention in the primary tumor. This amplifies tumor-derived T_RM_ cells in the lung and induces protection against metastasis, thus, overall suggesting a potential strategical approach to prevent metastasis [288].

T_RM_ cells are currently considered a valuable tool in tumor immunotherapy. For instance, checkpoint therapy enhanced T_RM_ formation in mice with melanoma [289]. Specifically, programmed cell death protein (PD)-1 inhibition in combination with central memory T cell transfer induced T_RM_ infiltration and subsequently inhibited tumor growth [289]. Similarly, PD-1 inhibition in human NSCLC-derived T_RM_ cells boosted their cytotoxic capacity against autologous tumor cells ex vivo [70]. Additionally, PD-1 blockade enhanced CD8^+^CD103^+^ T_RM_ cell proliferation in patients with melanoma, with higher cell numbers correlating with longer survival [290]. Hence, T_RM_ modulation appears to be a potent future approach to enhance cancer therapy efficacy.

## Research approaches for lung tissue-resident immunity assessment

Since the first identification of tissue-resident immunity, its assessment has been a challenge and calls for constant optimization of respective research approaches.

A first and likely the simplest method to address tissue-residence is the in vivo immune cell labelling (Fig. 4a). Intravenous staining distinguishes between immune cells in vasculature and those outside intact endothelium, e.g., in the lung parenchyma [291].

Another technique to label cells in the lung and track their movement in vivo is the photoconversion of one lung after thoracotomy (Fig. 4b). This method is possible for mice carrying the Kaede protein, a coral-derived fluorescent protein whose emission alters from green to red fluorescence after exposure to violet light. Photoconversion allows in situ labeling of Kaede protein-expressing cells in the lung [292].

Assessment of lung-resident immunity can also be achieved with parabiosis (Fig. 4c). Surgical generation of parabiotic pairs is the conjoining of two congenic mice, which share their blood circulation through newly developed vasculature within approximately one week. Through the blood circulation, recirculating lymphocyte populations equilibrate between the mice of the pair. Failure of an immune cell population to equilibrate between tissues in each mouse of the parabiotic pair indicates residence [293].

Finally, another powerful method to track tissue-resident immune cells is orthotopic lung transplantation, allowing both cell ingress and egress assessment (Fig. 4d). Tissue-resident cells are transplanted together with the organ in the recipient congenic mouse, thus, allowing one to discriminate between tissue-resident and circulating immune cells by the analysis of the congene in the transplant organ and in the periphery [66, 294].

## Sex-specific differences in tissue resident immunity

Sex differences in both innate and adaptive immunity are well documented in humans and mice [295]. However, insights into a potential sexual dimorphism of tissue-resident immunity remain sparse. Of note, sex differences in tissue-resident immune cells can be tissue- or organ-specific. For example, female mice have higher number of tissue-resident macrophages, T and B cells in naïve peritoneal and pleural cavities, compared to male ones [296]. Within macrophages, upregulated expression of TLRs, especially TLR2 and TLR4, was demonstrated and associated with a higher phagocytotic capacity in female mice. Among T cell subpopulations, CD4^+^ and CD8^+^ cells, but not Treg or γδ-T cells, were significantly increased in females compared to males. Additionally, increased lung-resident ILCs were observed in female compared to male mice in steady-state conditions [297, 298].

Further phenotypic and functional characterization of sex-specific differences in tissue-resident immunity is warranted to elucidate the mechanisms underlying the sex-specific manifestation of several respiratory immune diseases. For example, a sex bias in the incidence and severity of allergic asthma is well-established, with male and female predominance in childhood and adulthood, respectively [299]. Given the aforementioned role of tissue-resident immunity in asthma pathogenesis, sex-specific differences in tissue-resident immune cells and responses may – at least to some extent- account for this sex bias. Although the mechanisms driving the observed sex disparity in relation to asthma in general remain unclear, a crosstalk between immune cells and sex hormones seems to play a key role in this context and could likely apply in the case of tissue-resident immunity. Indeed, androgen signaling impacts ILC2 responsiveness while estrogen-mediated signaling influences macrophage polarization and therefore contribute to sex differences in allergic asthma [300, 301]. Additionally, estrogens promote mast cell degranulation thereby exacerbating asthma severity [297].

In conclusion, more studies are required in order to thoroughly characterize a potential sexual dimorphism in lung-resident immunity and uncover its role in respiratory health and disease. If obtained, such insights would pave the way for targeted optimized therapeutic approaches in a sex-dependent, highly personalized manner.
